# Transcriptome Profile of Thyroid Glands in Bile Duct Ligation Mouse Model

**DOI:** 10.3390/ijms23158244

**Published:** 2022-07-26

**Authors:** Danbi Jo, Hee Kyung Kim, Young-Kook Kim, Juhyun Song

**Affiliations:** 1 Department of Anatomy, Chonnam National University Medical School, Seoyangro 264, Hwasun 58128, Korea; danbijo0818@gmail.com; 2 Biomedical Science Graduate Program (BMSGP), Chonnam National University, Seoyangro 264, Hwasun 58128, Korea; 3 Division of Endocrinology and Metabolism, Department of Internal Medicine, Chonnam National University Medical School, Seoyangro 264, Hwasun 58128, Korea; albeppy@chonnam.ac.kr; 4 Department of Biochemistry, Chonnam National University Medical School, Seoyangro 264, Hwasun 58128, Korea; ykk@jnu.ac.kr

**Keywords:** thyroid gland, thyroid hormone (TH), bile duct ligation (BDL) model, hepatic encephalopathy (HE), RNA sequencing

## Abstract

Thyroid hormone (TH) contributes to multiple cellular mechanisms in the liver, muscle cells, adipose tissue, and brain, etc. In particular, the liver is an important organ in TH metabolism for the conversion of thyronine (T4) into triiodothyronine (T3) by the deiodinase enzyme. TH levels were significantly decreased and thyroid-stimulating hormone (TSH) levels were significantly increased in patients with liver failure compared with normal subjects. Among liver failure diseases, hepatic encephalopathy (HE) deserves more attention because liver damage and neuropathologies occur simultaneously. Although there is numerous evidence of TH dysregulation in the HE model, specific mechanisms and genetic features of the thyroid glands in the HE model are not fully understood. Here, we investigated the significantly different genes in the thyroid glands of a bile duct ligation (BDL) mouse model as the HE model, compared to the thyroid glands of the control mouse using RNA sequencing. We also confirmed the alteration in mRNA levels of thyroid gland function-related genes in the BDL mouse model. Furthermore, we evaluated the increased level of free T4 and TSH in the BDL mouse blood. Thus, we emphasize the potential roles of TH in liver metabolism and suggest that thyroid dysfunction-related genes in the HE model should be highlighted for finding the appropriate solution for an impaired thyroid system in HE.

## 1. Introduction

The thyroid gland produces major amine thyroid hormones (THs) such as thyroxine (T4) and 3,3′,5′-triiodo-L-thyronine (T3) [1]. The hormone T3 is considered the active form of TH because T3 can easily bind to thyroid receptors (TRs) with a higher affinity than that of T4 [2]. Furthermore, T4 is considered a pro-hormone and can be converted into T3 through enzymatic processing by local deiodinase enzymes [1,2].

Importantly, THs function as regulators of cellular signaling and cellular development [3] and important metabolic responses such as glucose metabolism, lipid metabolism, and cellular apoptosis [4]. In the central nervous system (CNS), TH affects multiple neuropathologies by binding to TRs that exist in numerous brain tissues [5,6,7]. The imbalance in serum TH levels is related to various diseases including metabolic diseases such as diabetes, obesity [8,9,10], cardiovascular disease [11,12], and liver-related disorders [13].

Moreover, TH contributes to the cellular and molecular function in liver failure diseases [14] and hepatic lipid metabolism [15]. Several epidemiological and clinical studies mention that general endocrine dysfunction caused by abnormal thyroid hormone results in cognitive dysfunction [16].

The liver is a critical organ in the effective conversion of T4 into T3 by type I deiodinase, which is a major enzyme responsible for 40% of T3 production [17]. Additionally, the liver is associated with TH excretion and synthesis of thyroid-binding globulin [17,18]. Furthermore, THs such as T3 and T4 modulate metabolic function in liver hepatocytes and contribute to liver metabolism, including bilirubin metabolism and hepatic circulation [14,19]. Thus, TH could reduce bile duct secretion and total bile acid size [20].

Clinically, patients with liver injury have TH abnormality, which is associated with liver dysfunction [21,22,23]. Recent studies have demonstrated that abnormal TH levels contribute to liver dysfunction [24] and lead to an imbalance of aspartate aminotransferase (AST) levels and elevated thyroid-stimulating hormone (TSH) levels in patients with liver cirrhosis [25]. Additionally, patients with non-alcoholic fatty liver disease show hypothyroidism because of lowered TH levels [15,26,27] and elevated TSH secretion [28]. Some studies have suggested that lower TH levels can accelerate the progression of liver fibrosis [29,30].

Although there is a lot of evidence that supports the strong relationship between liver function and TH metabolism, the mechanism and related genes are not fully understood.

Among liver diseases, hepatic encephalopathy (HE) is a very important liver disease as it shows both liver damage and brain dysfunction such as cerebral edema and astrocyte swelling through poor bile duct circulation [31,32,33].

Patients with HE show imbalanced levels of T3 and T4 [18,34]. One study stated that type-A HE shows a lower level of TH [35], while another suggested that patients with refractory HE showed hypothyroidism with ammonia dysmetabolism [36].

Although there is substantial evidence on the importance of TH function in patients with HE, significant evidence about the differential expression of several genes in the thyroid gland in an HE model compared to a normal model is lacking. Thus, we investigated the significantly altered coding RNAs and non-coding RNAs in the thyroid glands of a bile duct ligation (BDL) model as a HE mouse model [37] using RNA sequencing.

Next, we confirmed the effect of alteration of mRNA expression on thyroid function-related genes in the BDL model. In this study, we investigated the potential linkage of TH dysfunction in HE.

## 2. Results

**Figure 1:**
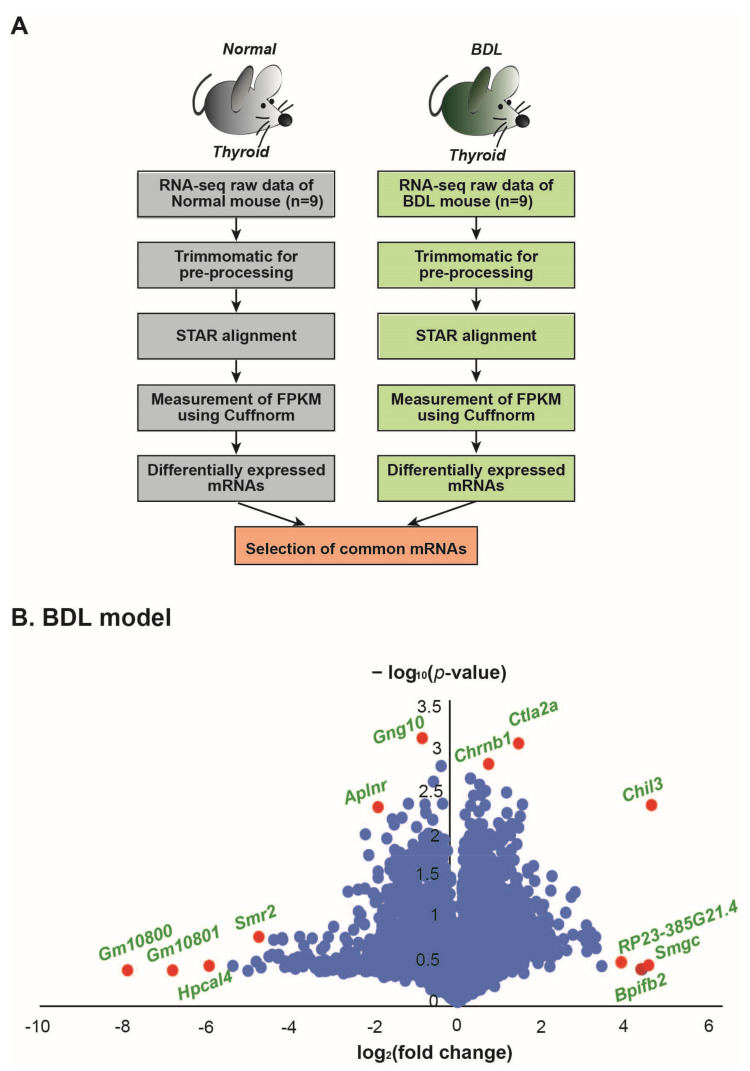
Analysis of transcriptomic data from the thyroid gland tissue of BDL mouse models. (**A**) Process for analysis of the transcriptome data. (**B**) Volcano plots of the BDL mouse model. The X-axis showed the log_2_-transformed fold change in each group and the Y-axis represents the −log_10_(*p*-value) value. Red dots indicate the genes with a significantly changed expression.

First, we investigated the transcriptome data of nine BDL mice’s thyroid glands and nine sham mice’s thyroid glands after RNA sequencing. After analyzing and comparing the RNA sequencing data (Figure 1A, see method), the genes with high expression and significant fold change in the thyroid glands of BDL mice were sorted and displayed by volcano plot graphs (Figure 1B).

In total, 393 genes in the thyroid glands of the BDL mouse model showed a significant change in the expression (*p* ≤ 0.05). As depicted in the volcano plot (Figure 1B), the expression of *Chrnb1*, *Ctla2a*, *Chil3*, *Bpifb2*, *Smgc*, *RP23-385G21.4*, *Gng10*, *Aplnr*, *Smr2*, *Hpcal4*, *Gm10801*, and *Gm10800* was significantly changed (Figure 1B).

**Figure 2:**
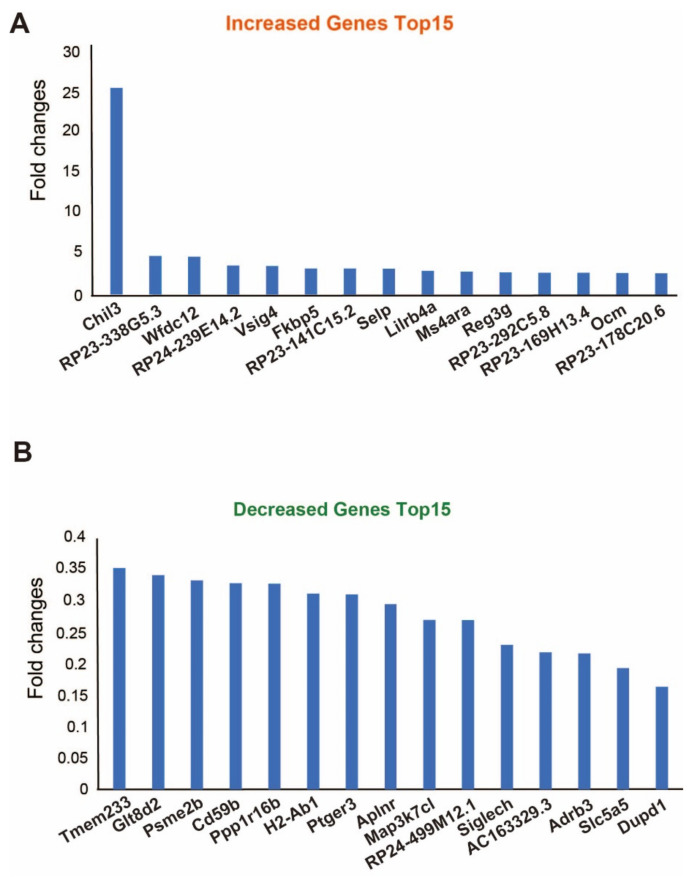
Selected genes with significant expression change in the thyroid gland tissue of BDL mouse models. (**A**,**B**) The common genes with a significant expression change in the thyroid gland tissue in BDL models. The graphs depict (**A**) the top 15 genes with increased expression and (**B**) the top 15 genes with decreased expression.

For the identification of the top 15 genes in the BDL mouse model and normal mouse model based on the fold changes, we chose 234 genes with more than log_2_(fold change) in the expression in the BDL mouse model (*p* < 0.05; Figure 2A). The identified genes were *Chil3*, *RP23-338G5.3*, *Wfdc12*, *RP24-239E14.2*, *Vsig4*, *Fkbp5*, *RP23-141C15.2*, *Selp*, *Lirb4a*, *Ms4ara*, *Reg3g*, *RP23-292C5.8*, *RP23-169H13.4*, *Ocm*, and *RP-178C20.6* (Figure 2A).

For the identification of the top 15 genes with a decreased expression in the BDL mouse model and normal mouse model based on the fold change, we chose 159 genes with less than log_2_(fold change) in the expression in the BDL mouse model (*p* < 0.05; Figure 2B). These were *Tmem233*, *Glt8d2*, *Psme2b*, *Cd59b*, *Ppp1r16b*, *H2-Ab1*, *Ptger3*, *Aplnr*, *Map3k7cl*, *RP24-499M12.1*, *Siglech*, *AC163329.3*, *Adrb3*, *Slc5a5*, and *Dupd1* (Figure 2B).

**Figure 3:**
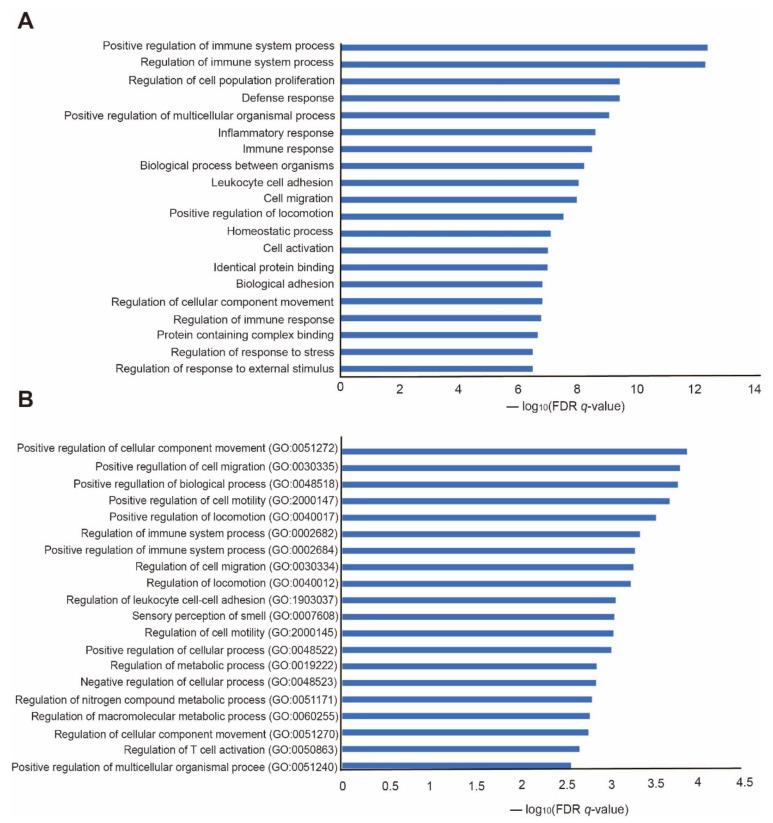
Functional analysis of genes with increased expression in BDL mouse groups. (**A**) KEGG pathway analysis for genes with increased expression in the BDL mouse group. The significantly changed pathways based on the false discovery rate (FDR) *q*-value are shown. (**B**) GO analysis for genes with increased expression in the BDL mouse group. The top 20 GO terms based on the FDR *q*-value are shown.

Additionally, to assess the cellular pathways associated with the genes with significantly changed expression, we conducted kyoto encyclopedia of genes and genomes (KEGG) pathway analysis using molecular signatures database (MsigDB) program (Figure 3A). It showed a significantly positive regulation of the immune system, cell proliferation, defense response, inflammatory response, immune response, leukocyte cell adhesion, cell migration, cell activation, and response to stress in the BDL mouse model group (Figure 3A). Next, we conducted gene ontology (GO) analysis to identify genes with significantly changed expression in the BDL group (The Gene Ontology Consortium, 2017) (Figure 3B). Genes with a significantly increased expression included those related to positive regulation of cellular component movement, cell migration, cell motility, leukocyte cell–cell adhesion, metabolic process, macromolecular metabolic process, and T-cell activation (Figure 3B).

Based on our data, we could identify alterations in the immune- and inflammatory-related genes in the thyroid glands of the BDL mouse model (see Discussion).

**Figure 4:**
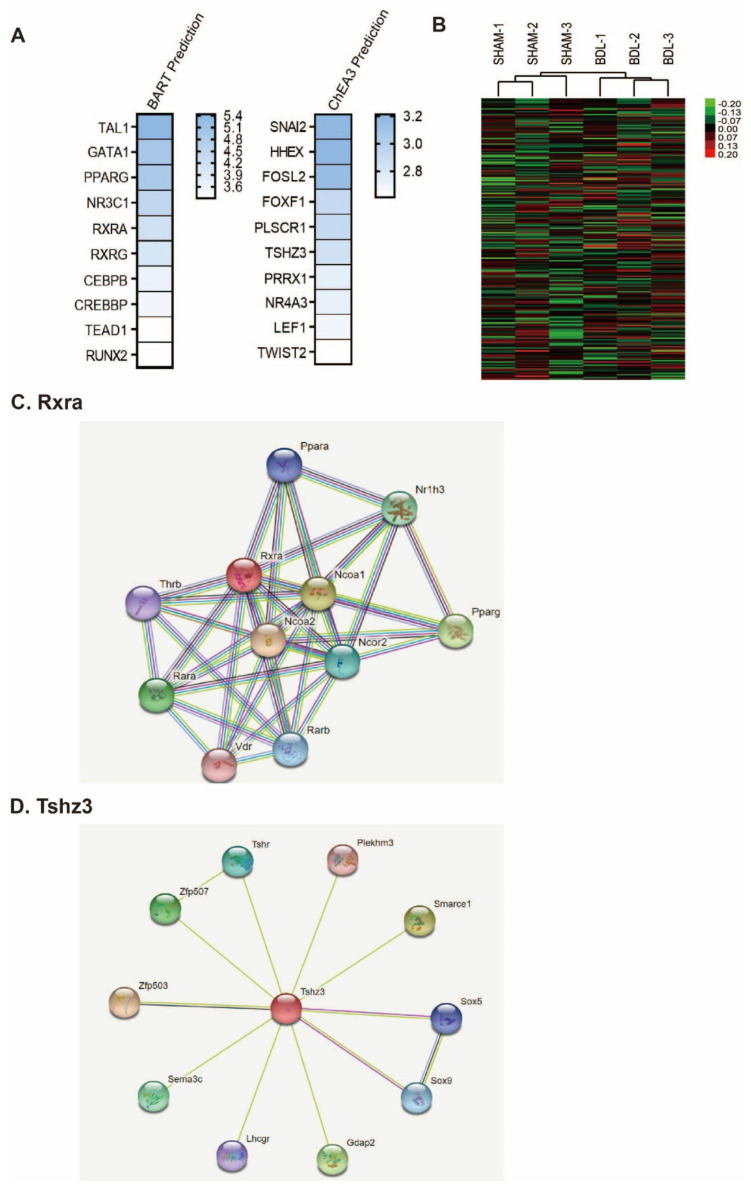
Transcriptional analysis of the common genes in the BDL mouse group. (**A**) BART prediction and ChAE3 prediction transcription factor analysis. (**B**) Heatmap of well grouping between the groups. (**C**,**D**) Signal networking data.

Furthermore, we conducted BART prediction and ChAE3 prediction for determining related transcription factors in the thyroid gland of those with liver failure (Figure 4A–D). BART prediction identified several transcription factors such as *TAL1*, *GATA1*, *PPARG*, *NR3C1*, *RXRA*, *RXRG*, *CEBPB*, *CREBBP*, *TEAD1*, and *RUNX2*, whereas ChAE3 prediction identified *SNAI2*, *HHEX*, *FOSL2*, *FOXF1*, *PLSCR1*, *TSHZ3*, *PRRX1*, *NR4A3*, *LEF1*, and *TWIST2* (Figure 4A). Figure 4B shows that genes in the same groups are well organized and that genes with a similar pattern were identified in sham 1, 2, and 3 groups and BDL 1, 2, and 3 groups (Figure 4B). We collected three thyroid glands and counted them as one sample. So, we conducted RNA sequencing by sampling a total of 9 thyroid glands by considering the collected 3 thyroid glands as one sample.

**Figure 5:**
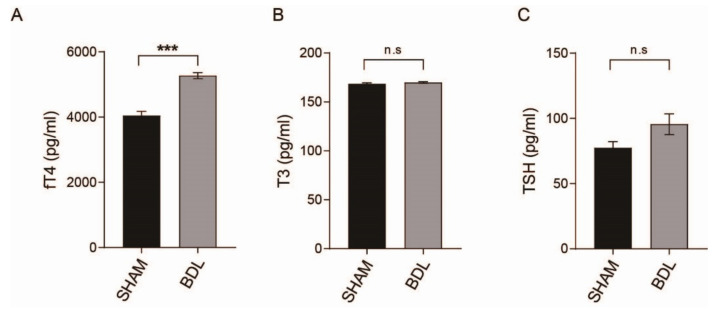
ELISA data. (**A**) ELISA to detect free T4, (**B**) T3, and (**C**) TSH in blood serum of sham and BDL mice. *p*-value *** is <0.001 and n.s means no significant.

Figure 4 data show that the thyroid gland after BDL surgery had altered TH secretion-related transcription factors such as retinoid X receptor alpha (*Rxra*) that is functionally connected with TH receptor beta (Thrb) and peroxisome proliferator-activated receptor (PPAR)-gamma (*PPARG*), and Teashirt Zinc Finger Homeobox 3 (*Tshz3*) that is functionally connected with thyrotropin receptor (*Tshr*) (Figure 4A,C,D). Additionally, we found a change in the immune response and inflammation involving transcription factors such as T-cell acute lymphoblastic leukemia 1 (*TAL1*) and CCAAT Enhancer Binding Protein Beta (*CEBPB*). Finally, we measured the level of free T4 (fT4), T3, and TSH in the serum of the BDL mouse and control mouse (Figure 5). fT4 level was markedly increased in the serum of the BDL mouse compared to that of the control mouse, whereas T3 and TSH levels were not significantly different between the BDL mouse and the control mouse (Figure 5A,B). TSH level was also not significantly changed, but an increased value was confirmed in the BDL mouse (Figure 5C).

## 3. Discussion

In this study, we also found high mRNA levels of *Chil3* in patients with papillary and anaplastic thyroid cancers [38]. Other studies have demonstrated that *Chil3* is repeatedly upregulated in those with thyroid cancer [39,40]. The expression of *Fkbp5* in the BDL mouse’s thyroid glands was considerably higher than that in the sham model’s thyroid glands. Considering recent evidence, *Fkbp5*, which interacts with heat shock protein 90, can accelerate the progression of papillary thyroid carcinoma [41,42].

Also, based on our results, *Selp* plays a critical role in metastasis mechanism and is associated with autoimmune thyroid diseases [43]. Increased expression of *Selp* can result in the development of Grave’s disease [44] and an abnormal level of *Selp* is presented in hyperthyroidism [45] and thyroid cancer such as papillary thyroid cancer [46,47,48,49].

Among decreased genes in the BDL mouse’s thyroid glands, *Slc5a5*, a sodium iodide symporter (NIS), is present in various organs such as the eye, pulmonary airway, and thyroid gland [50,51]. *Slc5a5* expression is controlled by TSH via the regulation of iodine uptake by thyroid follicular cells mediated by cAMP [50]. NIS activates the transport of iodide from the bloodstream into the thyroid gland [50,52,53]. Decreased *Slc5a5* gene expression is observed in patients with hypothyroidism [54,55] and papillary thyroid cancer [56].

Considering our GO analysis and KEGG data, the regulation of the immune system, cell proliferation, and inflammatory response was significantly affected in the BDL mouse model. Furthermore, based on BART and ChEA3 prediction data, we found considerable TH-related transcription factor alteration in *Rxra*, *Thrb*, *Tshz3*, thyroid receptor for TSH or thyrotropin, *TAL1* [57], and *CEBPB* (inflammation and leukocyte circulation related gene) [58].

BDL surgery in mice leads to an imbalance in TH, immune cell infiltration, inflammation in the thyroid gland, and a high risk of thyroid cancer. Previous studies have mentioned that abnormal TH secretion regulates the secretion of cytokines and ultimately controls inflammatory response and the immune system by modulating immune cell activity [59,60].

One study demonstrated that T3 and T4 could activate cytokine maturation and production through MAPK signaling [61]. Some studies have suggested that T3 could increase the proportion of activated dendritic cells and activated T lymphocytes by modulating specific cytokines such as IL-17 [62,63].

Other studies indicated that T3 and T4 are involved in the production of reactive oxygen species (ROS) in immune cells [59] and the migration and proliferation of immune cells under the inflammatory response [64].

We found the involvement of transcription factors such as *PPARG*, which is known to modulate the inflammatory response and cell migration in thyroid tissues [65,66].

A previous clinical study reported an abnormality in its expression in HE patients [35]. Patients with acute liver failure (ALF) and liver cirrhosis present a positive correlation between thyroid dysfunction [25] and thyroid cancer [67].

Our data showed an increased level of fT4 in the serum of the BDL mouse compared to the control mouse, but no significant data for T3, and a non-significant increase in TSH levels in the serum of the BDL mouse compared to the control mouse.

A recent study mentioned that acute liver damage results in an increased TSH level compared to that in normal subjects [23]. Previous studies demonstrated that liver failure such as HE leads to a low level of T3, an increased level of fT4, and an increased level of TSH [34,35,68].

In this study, there were several limitations for the interpretation of thyroid hormone change. We could not test serial thyroid hormone levels for work up of hormonal change to the progression of liver failure. Moreover, we could not confirm the invasion of inflammatory cells of the thyroid gland through histologic examination. On the 14th day after BDL, thyroid sampling for mRNA analysis and serum sampling for thyroid hormone were performed. GO analysis and KEGG data showed significantly increased regulation of the immune system and inflammatory response in the thyroid of the BDL mouse model. As the inflammation of the thyroid progresses, the release of thyroid hormone results in an increase in serum thyroid hormone levels. T4 is mainly produced and stored in the thyroid gland, therefore T4 is dominantly increased when the thyroid gland is destroyed. For this reason, fT4 is thought to increase in BDL mice. Based on these results, we emphasize the need for further study for understanding the imbalance of thyroid hormone in HE.

## 4. Materials and Methods

### 4.1. BDL Surgery and Thyroid Gland Sampling

Male C57BL/6 mice (aged 12 weeks) were purchased from Orient Bio (Seongnam, Gyeonggi-do, Korea) and used in our study. All mice were housed under handled conditions with a 12 h light/dark cycle and 21 °C temperature. The animals were provided free access to normal food and water. They were randomly divided into the control group or BDL group and then anesthetized using 5% isoflurane in mixed gas and maintained with 2% isoflurane during BDL surgery. The mice were placed on a thermostatic blanket, and their abdominal fur was shaved. Their abdomen was dissected using surgical scissors and after opening, the bile duct was ligated using a 5-0 silk suture. After BDL, the peritoneum and abdominal skin were closed with 5-0 silk sutures and sterilized with 70% ethanol. Thereafter, the mice were placed in their home cages and sacrificed 14 days after the BDL surgery. The sham mouse group received an operation to open and close the peritoneum. The thyroid glands of the mice (thyroid glands obtained from nine sham mice and nine BDL mice) were collected following cardiac perfusion with sterile saline, and total cellular RNA in the thyroid gland extracted using Trizol reagent (Invitrogen, Waltham, MA, USA). These were then stored at −70 °C until use. We collected three thyroid glands and counted them as one sample. All experiments were performed following the recommendations of the 1996 guidance for animal experiments established by the Animal Ethics Committee at Chonnam National University (CNU). The Animal Ethics Committee approved the protocol at CNU. Our study was conducted in compliance with the ARRIVE guidelines. The Animal Ethics Committee number is CNU IACUC-H-2022-8.

### 4.2. Analysis of RNA Sequencing Data

Among the RNA sequencing data obtained from the BDL and sham models, those with low-quality sequencing reads were well-groomed using Trimmomatic [69] (Figure 1A). The trimmed data sequences were arranged according to the mouse genome (mm10) using the spliced transcripts aligned to a reference (STAR) aligner [70]. Additionally, the Cuffnorm value was used to test normalized values of fragments per kilobase of transcript per million mapped reads (FPKM) given GENCODE annotation (Release M17, GRCm38.p6 [71] (Figure 1A). Transcripts with an average FPKM value of less than 1 or transcripts not detected in any sample were deleted from additional analysis (Figure 1A). A *t*-test was used to sort transcripts with a significantly different expression between the BDL group and sham group. Those with commonly altered mRNA expression between the BDL and sham groups were chosen for further functional analysis.

### 4.3. Functional Analysis of mRNAs

For the functional analysis of mRNAs, significant expression changes based on *p*-value < 0.05 were selected in both sham and BDL groups. Among them, those with changed expression in the same direction in both groups were selected. This filtering resulted in the identification of 393 significant genes in both groups (*p* < 0.05). Among them, we selected the top 15 genes with decreased expression and the top 15 with increased expression depending on fold changes. We used these 393 genes as common genes in both groups for KEGG pathway analysis and GO analysis using the Molecular Signatures Database [72].

### 4.4. Enzyme-Linked Immunosorbent Assay (ELISA)

For the measurement of thyroid hormone levels of the sham and BDL mouse groups, ELISA kits E-EL-M1153 (TSH) and E-EL-0122 (fT4) were purchased from Elabscience, and #K7422-100 (T3) was purchased from BioVision (BioVision, Milpitas, CA, USA) and used according to the manufacturer’s instructions. Briefly, standard or mouse serums were added to each well and biotinylated detection antibody working solution was immediately added to each well for 45 min at 37 °C. Solutions were decanted from each well and washed with 1X wash buffer three times. Horseradish peroxidase (HRP) conjugate working solution was added to each well and incubated for 30 min at 37 °C. Each well was washed five times, and substrate reagents were added to each well for 15 min at 37 °C. Finally, stop solution was added to each well and absorbance was measured with a microplate reader (BioTek, Winooski, VT, USA) at 450 nm. The concentration of hormones was calculated according to standard curves and the results are presented as the concentration (pg/mL).

### 4.5. Statistical Analysis

Data are shown as the mean ± standard error of the mean (SEM). The statistical comparisons between the sham and BDL groups were made with an unpaired *t*-test using SPSS 10 version software. A *p*-value < 0.05 was considered statistically significant.

## 5. Conclusions

In conclusion, we investigated the gene expression alterations in the thyroid gland tissues in a HE mouse model. Our data showed the possibility for increased immune cell migration and increased inflammatory response in the thyroid gland of HE compared to the normal thyroid gland. Even though we have some limitations, our study highlights the possibility that HE in patients is related to thyroid inflammation.

Thus, we suggest that the modulation of thyroid hormone level and thyroid inflammation-related genes may be new therapeutic approaches to improve pathologies in HE.

## Data Availability

Not applicable.
